# Caregivers' role in using a personal electronic health record: a qualitative study of cancer patients and caregivers in Germany

**DOI:** 10.1186/s12911-020-01172-4

**Published:** 2020-07-13

**Authors:** Aline Weis, Sabrina Pohlmann, Regina Poss-Doering, Beate Strauss, Charlotte Ullrich, Helene Hofmann, Dominik Ose, Eva C. Winkler, Joachim Szecsenyi, Michel Wensing

**Affiliations:** 1 Department of General Practice and Health Services Research, University Hospital Heidelberg, Im Neuenheimer Feld 130.3, 69120 Heidelberg, Germany; 2 Ethics and Patient-Oriented Care, National Center for Tumor Diseases (NCT), Im Neuenheimer Feld 460, 69120 Heidelberg, Germany; 3 Department of Family and Preventive Medicine, University of Utah, 375 Chipeta Way, Salt Lake City, UT 84108 USA

**Keywords:** Patient portals, Caregivers, Qualitative research, Patient participation, Gastrointestinal neoplasms

## Abstract

**Background:**

Particularly in the context of severe diseases like cancer, many patients wish to include caregivers in the planning of treatment and care. Many caregivers like to be involved but feel insufficiently enabled. This study aimed at providing insight into patients’ and caregivers’ perspectives on caregivers’ roles in managing the patient portal of an electronic personal health record (PHR).

**Methods:**

A descriptive qualitative study was conducted comprising two study phases: (1) Usability tests and interviews with patients with cancer and caregivers (2) additional patient interviews after a 3-month-pilot-testing of the PHR. For both study parts, a convenience sample was selected, focusing on current state of health and therapy process and basic willingness to participate and ending up with a mixed sample as well as saturation of data. All interviews were audio-recorded, pseudonymized, transcribed verbatim and qualitatively analyzed.

**Results:**

Two main categories emerged from qualitative data: ‘Caregivers’ role’ and ‘Graduation of access rights’ – consisting of four subcategories each. The interviewed patients (*n* = 22) and caregivers (*n* = 9) felt that the involvement of caregivers is central to foster the acceptance of a PHR for cancer patients. However, their role varied from providing technical support to representing patients, e.g. if the patient’s state of health made this necessary. Heterogeneous opinions emerged regarding the question whether caregivers should receive full or graduated access on a patient’s PHR.

**Conclusions:**

In order to support the patient and to participate in the care process, caregivers need up-to-date information on the patient’s health and treatment. Nevertheless, some patients do not want to share all medical data with caregivers, which might strain the patient-caregiver relationship. This needs to be considered in development and implementation of personal health records. Generally, in the debate on patient portals of a personal health record, paying attention to the role of caregivers is essential. By appreciating the important relationship between patients and caregivers right from the beginning, implementation, of a PHR would be enhanced.

**Trial registration:**

ISRCTN85224823. Date of registration: 23/12/2015 (retrospectively registered).

## Background

The diagnosis of a severe disease, such as cancer, means a fundamental change for patient’s life and life plans in most cases. Professional service providers, i.e. physicians or nurses, can support this coping process to a limited extent only. Thus, informal caregivers play an important role, for example by supporting patients to manage a serious disease and possible consequences. Here we focus on the role of informal care providers in the use of personal electronic health records (PHR) because previous studies already have shown that they might contribute possibilities to improve informal caregiver involvement e.g. by bridging distances between family members living away from each other [1].

These informal caregivers include family members such as spouses, parents, children, siblings and others (e.g. friends, neighbors). Roles of caregivers in supporting patients are multifarious and range from being there to talk to up to supervising patients’ symptoms and wellbeing outside the hospital or professional care setting or supporting patients in treatment decisions [2]. However, research has shown that most caregivers are not well-prepared to fulfill those tasks. Several studies stressed that more information and support would be necessary to support caregivers when they take on responsibility in a patients’ treatment and care [2–5]. Kent et al. (2016) stated that one step to meet those needs might be to enable caregivers to access the patients’ health-related information via electronic health records [2]. In cancer care, patients and caregivers face a complex treatment that is characterized by a lot of medical appointments, resulting in many documents and paperwork that need to be managed. To this end patient portals are meant to serve as an access to this information stored within a PHR. Generally, the availability of personal health information and the document management via such patient-controlled portals is becoming increasingly appreciated because it could also enhance patient involvement in treatment [6, 7].

Considering the important role of caregivers in cancer care, many patients and caregivers would like to have the opportunity to use a patient portal together [8, 9]. Apart from the context of pediatric application scenarios [10], until now only a few online patient portals allow such shared accesses for adults [1, 11]. Despite the growing relevance of PHRs and caregivers’ access to them, there is so far no consensus on the clear role for caregivers in the use of a PHR. Therefore, one objective of the underlying research project “Information Technology for patient-oriented health care in the Rhine-Neckar region (INFOPAT)” was not only to develop a patient-centered PHR by involving patients themselves but also to illuminate the caregivers’ attitudes and opinions regarding this subject [8, 12–17].

The study aimed to answer the following research questions:

1) What role might caregivers play in the use of a PHR from the point of view of patients and their caregivers?

2) How might caregivers get involved into managing a patient’s PHR?

## Methods

To gain insights into the caregivers’ role in using a PHR a qualitative study design was chosen. It comprised of two phases, which were partly overlapping. The first phase was a pre-implementation study serving as an initial evaluation of the PHR-prototype developed in the project context. The second phase was a feasibility study that further investigated to what extent an implementation of the PHR is feasible for patients under real care conditions.

**Fig. 1:**
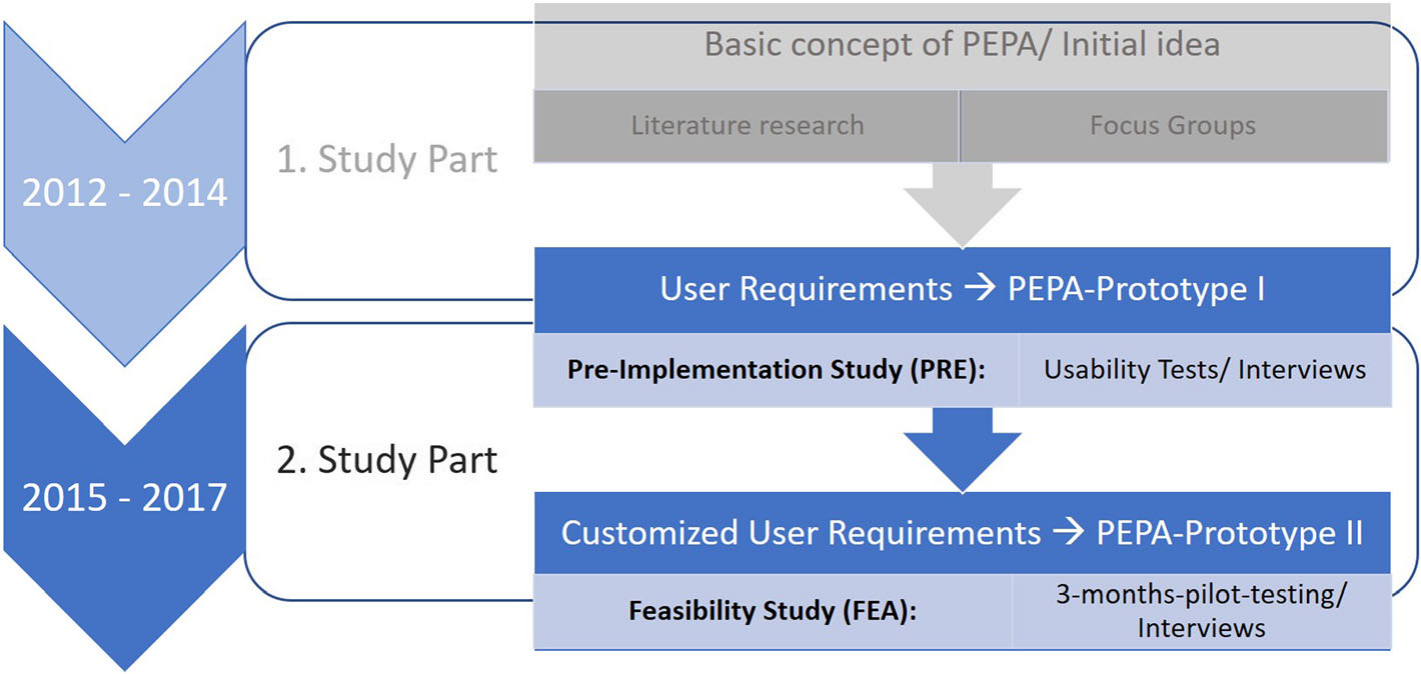
Study parts of the project and their interconnection

The study presented in this manuscript is a descriptive qualitative study which was part of a research project running for several years in different parts. Figure 1 shows the different study parts and their interconnection. The data dealt with in this manuscript stems from the second study part. However, the interview guide as well as the initial search grid (category system) used for data analysis resulted from the first study phase and the focus groups that were performed in this stage of the project.

To ensure trustworthiness of chosen methods and subsequent findings, appropriate strategies were applied with the aim of generating rich description of findings with sufficient details to enable the evaluation of the extent of transferability. Strategies included triangulation of data sources and peer debriefing to critically discuss methodology, data analysis and interpretations within the research team as well as with skilled qualitative researchers who were not directly involved in the research project [18]. The reporting of methods and findings follows the COREQ checklist recommendations [19].

### Research setting

The undertaken research was part of a study dealing with the development and pilot-implementation of a PHR within the project “Information Technology for patient-oriented health care in the Rhine-Neckar region” (INFOPAT) [8, 14–17, 20]. The overall aim of INFOPAT was to establish structures and processes that enable integrated and cross-sectoral care of chronically ill people. One central step for achieving this goal was the establishment of a cross-institutional PHR called “PEPA”.

### Sampling and recruitment

The recruitment of participants was conducted through the National Center for Tumor Diseases (NCT) in Heidelberg, Germany and the INFOPAT study team (Department of General Practice and Health Services Research, University Hospital Heidelberg). Potential participants were contacted during their NCT visits for example while receiving outpatient chemotherapy. For both study parts, a convenience sample was selected, focusing on current state of health and therapy process as well as basic willingness to participate. Given that potential recruits were gravely ill persons, it was not feasible to follow a purposive sampling strategy with regards to variety of age, gender, or education and as usual in qualitative research, no formal sample size was calculated. Nevertheless, we ended up with a mixed sample and reached saturation of data.

#### Pre-implementation study

Between November 2015 and December 2016 an oncologist from NCT screened patients receiving chemotherapy at NCT with a diagnosis of gastrointestinal tumor diseases (i.e., ICD-10 C18, C19, C20, C16, C23.9, C24.0, or C24.1) and caregivers accompanying other patients with the same diagnoses to NCT-visits. Patients with a recent diagnosis as well as those who had been in treatment for some time could participate. Caregivers were defined as persons playing a role in the management of patients’ disease (e.g. family, friends or neighbors). Patients and caregivers recruited for participation in the pre-implementation study were not necessarily in a personal relationship with each other. Inclusion criteria were an age of at least 18 years, full legal capacity and persons being [fluent in German]. Patients and caregivers not meeting these criteria or those suffering from severe acute psychiatric, behavioral or psychological disorders or dementia were excluded. Persons meeting all defined criteria received detailed information via mail and telephone and were included in the trial by scheduling in-person sessions to obtain written informed consent and complete the usability test and interview.

#### Feasibility study

Between July and August 2016, a random sample of 17 patients was screened by the same oncologist from NCT for participation in the study which took place between August and November 2016. In addition to the inclusion and exclusion criteria of the pre-implementation study, only patients with access to a computer with internet connection and participating in the study-specific training were eligible for participation.

### Data collection

#### Pre-implementation study

Since this study phase aimed to simulate PEPA-use under real-world conditions a usability test was developed that simulated activities which would occur if PEPA was used in everyday life. Therefore, different scenarios were built up and pilot-tested for patients and caregivers, for example dealing with registration, home page, document management and authorization administration. Due to technical problems with the PEPA prototype two usability tests with caregivers could not be conducted per protocol, but were replaced by a static presentation of the respective PEPA-features.

Following the usability test, which lasted for an average of 37 min and took place either in the NCT or the Department of General Practice and Health Services Research, face-to-face interviews were conducted using a semi-structured interview guide. The guide was based on a literature review and focus groups which were performed prior in the same project context [8, 13, 16]. On average, the interviews lasted 25 min. All interviews were conducted by AW or SP, documented in field notes and were audiotaped. At the request of participants, relatives could be present at the usability tests and interviews. Participants completed a pseudonymized sociodemographic questionnaire. Patients’ usability tests and interviews took place between December 2015 and February 2016 and caregivers’ appointments between April 2016 and December 2016.

#### Feasibility study

Patient interviews within the feasibility study took place after three months of pilot testing the PEPA between November 2016 and January 2017. On average, the duration of interviews was 50 min and all conversations were recorded on audiotape. Due to patient preferences, two interviews were conducted via telephone, the other interviews took place at the Department of General Practice and Health Services Research. Within the study-specific training prior to the start of the three months pilot testing phase, participants completed a brief pseudonymized sociodemographic questionnaire.

### Data analysis

Audio recordings from both study phases were pseudonymized and fully transcribed by trained staff. Subsequently, a qualitative content analysis was performed and pseudonymized quotes were extracted from the transcripts and edited to improve readability. Data analysis used a complex stepped process that applied a deductive initial search grid pre-defined by two researchers (AW, BS) followed by the inductive development of categories emerging from the data.

**Fig. 2:**
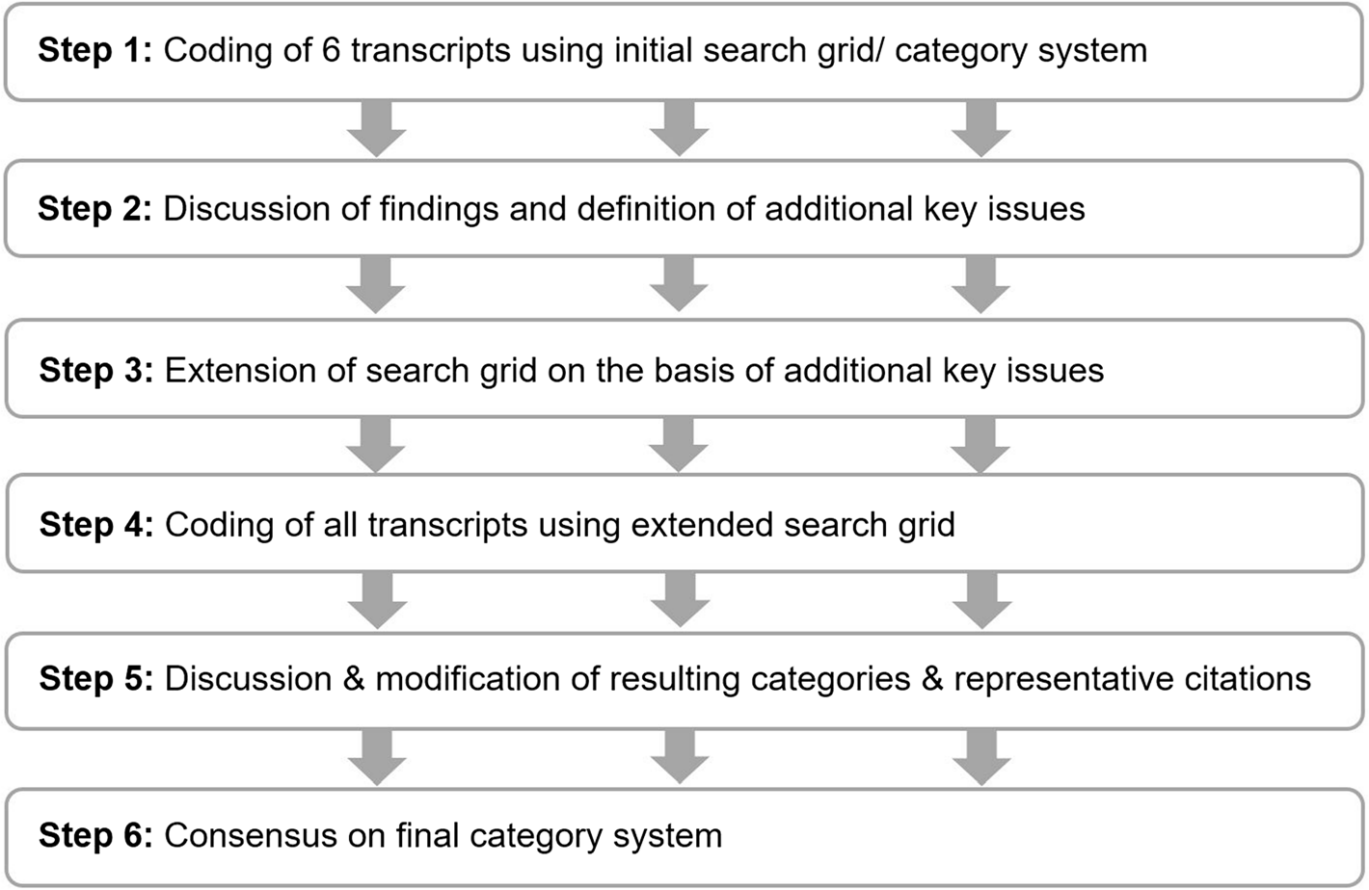
Step-by-step conduction of data analysis

Figure 2 shows the complete analysis process. The initial search grid (category system) resulted from preliminary analyses within the same project context [8, 13, 16]. Six transcripts (Pre-Implementation Study: 2 patients, 2 caregivers; Feasibility study: 2 patients) were reviewed independently by the first author (AW) and a co-author (BS) using the category system. Following that, findings were discussed between those two researches and additional key issues not yet covered by the search grid were identified and added to it. After summarizing and labelling all key issues as codes, the codes were sorted into main and subcategories and linked with representative citations from the original transcripts. The resulting categories were discussed and further modified within an interprofessional researcher team (AW; CU; SP; RPD) until a consensus on the final category system was achieved. AW, SP and RPD also engaged in the interview preparation and conduction while CU and BS were not involved in this process. To ensure a comprehensive view on the data, the team was made up of a wide range of experienced specialists from different disciplines (Social Sciences, Pharmacology, Health Sciences) [21]. For organizing and coding the data, the software Atlas.ti (Version 7.5.18, Scientific Software Development GmbH, Berlin, Germany) was used.

## Results

### Description of sample

In total, the interview transcripts of 31 participants from the pre-implementation study and the feasibility study were analyzed within this work. Table 1 presents the characteristics of the interview participants distributed among three different groups: (1) patients participating in the pre-implementation study (Pat_PRE/ *n* = 11), (2) relatives participating in the pre-implementation study (PAT_Ang/ *n* = 9), and (3) patients who participated in the feasibility study (Pat_FEA/ n = 11). The mean age across all participants was 54 years and 55% were female (*n* = 17).

**Table 1:** Overview of Study Participants

Participant group	ID	Sex	Age	Level of education	Comments
**Pre-Implementation Study/ Patients**	PRE_P_2	f	55	Secondary school leaving certificate	Friend attended Usability Test & Interview
	PRE_P_3	f	66	Secondary school leaving certificate	Spouse attended Usability Test & Interview
	PRE_P_4	m	48	University degree	Spouse attended Usability Test & Interview
	PRE_P_5	m	60	Lower secondary school leaving certificate	
	PRE_P_6	f	47	High school graduation	
	PRE_P_9	m	63	Lower secondary school leaving certificate	
	PRE_P_13	m	48	Secondary school leaving certificate	Spouse attended Usability Test & Interview
	PRE_P_14	f	67	Lower secondary school leaving certificate	Spouse attended Usability Test & Interview
	PRE_P_15	m	47	Secondary school leaving certificate	
	PRE_P_16	m	53	University degree	
	PRE_P_17	f	68	University degree	Child attended Usability Test & Interview
**Total PRE_P n = 11**					
**Pre-Implementation Study/ Caregivers**	PRE_C_01	f	53	Secondary school leaving certificate	Caregiver’s relation to the patient: Child
	PRE_C_04	f	46	Secondary school leaving certificate	Caregiver’s relation to the patient: Spouse
	PRE_C_05	m	67	Lower secondary school leaving certificate	Caregiver’s relation to the patient: Spouse
	PRE_C_14	f	52	Secondary school leaving certificate	Caregiver’s relation to the patient: Spouse
	PRE_C_15	f	64	Secondary school leaving certificate	Caregiver’s relation to the patient: Spouse
	PRE_C_16	f	36	University degree	Caregiver’s relation to the patient: Child
	PRE_C_17	f	51	Secondary school leaving certificate	Caregiver’s relation to the patient: Parent
	PRE_C_20	m	67	Lower secondary school leaving certificate	Caregiver’s relation to the patient: Parent
	PRE_C_22	m	33	Secondary school leaving certificate	Caregiver’s relation to the patient: Spouse
**Total PRE_C n = 9**					
**Feasibility Study/ Patients**	FEA_P_2	f	27	High school graduation	
	FEA_P_5	f	53	Lower secondary school leaving certificate	
	FEA_P_6	m	49	University degree	Patient took part in PRE as well
	FEA_P_10	f	61	Secondary school leaving certificate	
	FEA_P_11	m	56	Lower secondary school leaving certificate	
	FEA_P_12	f	44	University degree	
	FEA_P_13	f	55	Secondary school leaving certificate	
	FEA_P_15	m	61	University degree	
	FEA_P_21	m	64	University degree	
	FEA_P_27	f	43	Secondary school leaving certificate	
	FEA_P_30	m	57	University degree	
**Total FEA_P n = 11**					

Table legend: PRE = Pre-Implementation Study / FEA = Feasibility Study / P = Patient / C = Caregiver

### General findings

Referring to the underlying research questions, two main categories emerged from qualitative data as shown in Table 2. Furthermore, a total of eight subcategories – four in each main category – were identified (Table 2).

**Table 2:** Overview of main and subcategories emerged from qualitative data

Main categories	Subcategories
Caregivers’ role (CR)	Caregivers’ tasks and responsibilities (CR1)
	Realization of caregivers’ access (CR2)
	Caregivers as transmitters for emergency data (CR3)
	Comparison with patient decree/ health care proxy (CR4)
Graduation of access rights (GA)	Opportunities to access a patient portal (GA1)
	Full access for caregivers (GA2)
	Question of trust (GA3)
	Patients’ privacy (GA4)

The first section below will elaborate on the role caregivers actually fulfilled and which tasks they might take over in a PEPA-usage scenario. The results revealed different ways to realize a caregiver’s proxy access. This might be needed if a patient wants to transmit complete patient portal management or certain tasks to someone else – either for a limited period of time or longer-term. The second section focuses on the question whether caregivers should receive full or graduated access on a patient’s PEPA. Both subchapters contain a table with subcategories that are further explained in the text (see references referring to caregivers’ role (CR 1–4) in Table 3 and referring to graduation of access rights (GA 1–4)) and representative citations in Table 4.

**Table 3:** Subcategories and representative citations referring to caregivers’ role (CR) in a PEPA-scenario

Subcategory	Selection of representative citations
CR1) Caregivers’ tasks and responsibilities	*“Well, I think the advantage for me would definitely be that I could just authorize another person. Because I often had the situation that I needed some documents and then I said ‘Yes, Mom, drive home quickly, the folder is there and there, third page right, yes.’ And so, I can simply authorize her and she could then look in up. That would be an important advantage for me.” (FEA_P_2)* *“That’s why it’s so important - that there are external access possibilities. That you don’t have access alone and if you are lying in bed then you are cut off from the world. […] It must not be like this.” (FEA_P_12)*
CR2) Realization of caregivers’ access	*“[…] We would probably do that with one access, because we keep no secrets from each other and would simply use it together. If he [the patient] couldn’t, I’d probably have to manage that, maybe he’d take a look sometimes, but usually it’s something about what he says, that he wants to have as little as possible to do with it personally […] the good days should be good days - and why waste time on something like that?!” (PRE_C_4)* *“Yes, I granted my son access, I gave him my login data.” (FEA_P_10)* *“Let’s say, a patient is busy getting well. That doesn’t leave much time or patience for such a portal. I’m going to say that as a husband, I took on the role of managing all the documents by and large.” (PRE_C_22)*
CR3) Caregivers as transmitters for emergency data	*“It might be necessary, yes?! If you are in a hospital and you can’t do it or something, then it would probably be important that someone does it. [...] You should release that separately, I think. Not everything but again a selection. [...] The emergency dataset might be enough, I don’t know.” (PRE_P _15)*
CR4) Comparison with patient decree/ health care proxy	*“[…] if I make a living will now, I have to use someone who ultimately makes the decision. […] I’m not entering someone I don’t have full confidence in or with whom I haven’t discussed what would happen if…” (PRE_C_17)*

**Table 4:** Subcategories and representative citations referring to graduation of access rights (GA)

Subcategory	Selection of representative citations
GA1) Opportunities to access a patient portal	*“Why should I be the only one who knows what I’ve got? I don’t see any use in it. No, so my husband also looked at it [the PEPA-content].” (FEA_Pat_27)* *“In my PEPA no one else is supposed to be able to do anything but the people who are entitled. So, the doctors who upload, and me. Yes, maybe only reading permission [for the son].” (FEA_Pat_30)* *“I think it’s heavily dependent on the patient. For my part, I have full confidence in my parents. I would give them my login data, would tell them ‘You can look at anything’. But I could also imagine that there are other patients who would like to have access management options and be able to say ‘Yes, my parents may look in here and there if necessary. But, I don’t know, my gynecological findings are none of their business.” (Patient statement in Interview with PRE_C_16)*
GA2) Full access for caregivers	*“Everything! Because in my opinion, who can look into that file is a person of trust. [...] So that’s how I would do it, I wouldn’t say ‘Well, no! This content is restricted and that is restricted!’ But either I do trust in someone and he can look into the PEPA or I don’t and he cannot [...] look into it.” (PRE_C_4)* *“It’s like this, you have to imagine, my mother doesn’t even read the doctor’s letters. […] She doesn’t really want to know everything [...] and in fact I do want to know everything. [...] I also have a care directive, so if anything should happen, I have access everywhere [...]. [...] Someone has to know.” (PRE_C_1)*
GA3) Question of trust	*“I wouldn’t consider it useful if I had other permissions. Because, do I need someone, if I don’t trust him and give him the same permissions. That should be the person I really trust the most. Who really has insight.” (PRE_C_17)*
GA4) Patients’ privacy	*“I think everyone is entitled to privacy – also in the family. And that should be respected and that means – yes – […] If he would manage it himself and say, ‘You have nothing to do with it’, then I would respect that. But then he has to do it with all the consequences.” (PRE_C_15)*

### Caregivers’ role in a PEPA-scenario

The interviews showed that in all cases caregivers played a role in dealing with the patients’ disease, but the extent to which they were involved differed from case to case. Generally, their tasks included communication with physicians, help in understanding findings and further medical information, taking charge of filling prescriptions or forwarding sick notes as well as discussing therapy decisions, e.g. regarding prescribed medications, with the patient. Many caregivers also supported the patient in managing health-related documents.

Interviewees named many other possible tasks that caregivers might take over in a PEPA-usage scenario (CR1): Data management support, data transfer in case of emergency, technical support or support in case of comprehension difficulties. Data management support included setting and retrieving information from a patient’s PHR. Data transfer referred to the possibility to give physicians or other health care professionals access to information contained in the PHR, e.g. an emergency dataset consisting of diagnoses, allergies, current medication etc.. Furthermore, patients who are not computer-savvy stated that they would probably ask their children or partners for support on technical matters, e.g. login into the patient portal, managing documents or access rights. If patients had problems in understanding medical terms in the documents within the PHR they stated that they would also ask for support of their caregivers.

Another aspect named by caregivers was that the PEPA might serve as an assistance tool for caring relatives. One interviewed caregiver for example would like to have more information on how to support the patient – e.g. by reminding him to drink sufficient amounts of water.

Regarding the realization of a caregiver’s proxy access for the PEPA different viewpoints from patients and caregivers manifested (CR2). On the one hand, there were many interviewees who saw a PEPA as a shared project which will be managed together or – if the patient feels unwell or does not want to take care of it himself – through the caregiver. Therefore, some of them said that they would simply use the same account by handing over patients’ username and password. This way of managing a patients’ PEPA was mostly seen as easiest option to include a caregiver as representative. On the other hand, there were also some caregivers who stated that they would see the main responsibility for managing a patient’s PEPA on their side, because they wanted to take that burden from the patient.

However, some interviewees also brought up the issue of how they might work together successfully on a patients’ PEPA. They stated that it might not be self-explanatory how filenames and data storage within a PEPA must be organized in order to make all content detectable for every user. This topic also was relevant when it came to physicians and other healthcare providers searching for information in a PEPA.

It also was discussed that in some contexts it might be reasonable to have a separate account for caregivers. Especially, if the caregiver does not live in the same household as the patient this was considered helpful. A reason for this inter alia was the TAN-list which is obligatory to log-in into the PEPA. With two separate accounts each user would receive their own TAN-list to facilitate handling despite a geographical distance.

Another idea, which was brought up by one patient, was that caregivers should be able to pass on an emergency dataset in cases where the patient cannot manage physicians or other healthcare professionals’ access to his PEPA himself (CR3). When discussing a caregiver’s role in using a PEPA in two cases a comparison was drawn with topics like patient decree or health care proxy (CR4). Connected with the role caregivers already fulfill or which they could take over in a PEPA-scenario, the question was discussed which set of information should be available to them.

### Graduation of access rights

The question whether caregivers should receive full access on a patient’s PEPA or if graduated access would be sufficient, was discussed controversially by interview participants.

Patients expressed different expectations regarding their caregiver’s access opportunities (GA1). Some had the opinion that their caregiver was deeply involved into their treatment process anyway and therefore should also have full access to PEPA content and functionalities. Nevertheless, patients named different constellations in which it could be reasonable to graduate caregivers’ access such as not wanting to share sensitive information, or in order to safe caregivers from negative information like end-stage diagnoses. In only one case a patient said that he would not necessarily want one of his relatives to have access to his PEPA, and if they would have access it should only be a reading permission so that they are not able to add or change PEPA-content. When speaking about the need for gradable access rights patients and caregivers mostly covered this topic with reference to hypothetic constellations and underlining that they would generally confide all disease related information to their trusted caregivers.

Eight of nine interviewed caregivers voiced the opinion that they should have the same access rights as the patient (GA2). This included full insight into the data contained in the PEPA as well as managing access rights for health care professionals. Some caregivers even stated that the whole concept of a PEPA representative only makes sense if the caregiver has full access.

In three cases situations were depicted in which the patient himself did not really want to get into too much detail of disease specific information. Therefore, caregivers already took over the job to store and manage disease related data and consequently would also care for the patient’s PEPA rather self-sufficiently.

A topic which was brought up in several interviews was the question of trust in the context of using a patient’s PEPA together with a caregiver (GA3). Many patients and especially interviewed caregivers stated that it was self-evident that they would share the PEPA-content as well as access management rights with one or even more trusted caregivers. Most of the caregivers said it was natural to them that they would receive full access rights from the patient they care for.

Nevertheless, nearly all of them were aware of possible situations in which it might be helpful to have the opportunity to confer graded accesses (GA4). One caregiver even said that she wished to have full access on her husband’s PEPA but that she probably would give him only restricted access if she had a PEPA herself in order to not burden him in case she would have health related problems herself. Apart from this case there were other exceptions named, in which no full access would be permitted. Some patients and caregivers said that they would probably not want their children to have access on every detail of their PEPA. Only one caregiver clearly underlined that she would respect her spouse’s wish for privacy referring to using a PEPA.

## Discussion

On the question of caregivers’ role in the use of a PHR, study participants stated that involving caregivers in a PHR-scenario is an important step to generate a useful solution for managing disease-related information. The results indicate that there are several possible ways to involve caregivers because the level of caregiver involvement and the range of caregiver tasks differs from case to case. Especially with regards to the question whether a representative should be able to see each and every detail of a patients’ PHR or just contents authorized by the patient participants’ opinions differed. Therefore, tailoring the technical solution to individual patients and caregivers needs and preferences is essential. The following paragraphs discuss the role of relatives in a PEPA scenario and the graduation of access rights.

1. Caregivers’ role in a PEPA-scenario

In accordance with the presented results referring to caregivers’ role in cancer contexts, previous studies have demonstrated that caregivers take over a broad range of tasks in order to support their loved ones [2, 4, 22]. Apart from their supporting role for the patient, Laryionava et al. (2018) stated that caregivers supporting a cancer patient can be characterized as “second patients” because they are affected by patient’s disease in many ways and [23], therefore, need specific-tailored support, e.g. for coping with emotional distress.

The findings of the conducted interviews referring to sharing information between patient and caregiver are also confirmed by studies focusing on involvement of family members in shared decision making processes [24–28]. Shared decision making can only be successful if the relevant information is available [26]. This applies not only to the patient, but also if caregivers should be involved in the decision-making process. Therefore, PHR’s can serve as an information platform which brings together information for patients, caregivers and healthcare professionals [1].

As the results of this study show, caregivers often are dependent on receiving information on the patient’s state of health for being able to give support accordingly and participate in the care process. This becomes even more important in light of demographic change and the tendency to transfer healthcare increasingly into the homecare sector with informal caregivers taking over more responsibility [29]. As Lund et al. (2014) stated, primary and very close caregivers are deeply involved into caregiving tasks and connected worries, but also those caregivers being more distant are affected to a comparable extend [30].

Referring to the realization of proxy accesses, a lot of prior work was done within other fields like Human-Computer Interaction. Regarding the use of one single account for patient and caregiver, the results of the present study reflect those of Matthews et al. (2016) who found that household members often shared devices, such as smartphones and tablets, or accounts [31]. Another study on the use of single user device access in couples showed that even if they knew each other’s passwords (e.g. for e-mail accounts) they respected their partner’s privacy and did not use them to log in [32]. These results further support the idea of the caregivers and patients in this context who wanted to have the opportunity to log into the PHR just in case the patient was not willing or able to because of a health status deterioration.

In line with the results of the present study, information and communication technologies can help individuals to feel closer to each other despite of long distances [32]. Therefore, the inclusion of caregivers within a PHR-scenario also can contribute to improving patient’s and caregiver’s sense of connection and wellbeing. However, to include caregivers in the use of a patient portal, they already need to play a role within the development of PHR solutions. User-centered development processes therefore need to be strengthened.

(2) Graduation of access rights.

Since health-related information is highly sensitive, it is understandable that someone might not wish to share parts of it – even with persons very close. This finding supports previous research in the field of informal caregiving in the context of severe diseases or palliative treatment [33, 34]. Mazurek et al. (2017) figured out that nearly every interviewee they spoke to was fond of having the opportunity to administrate access to personal data stored in their devices. They also confirmed that it can be quite difficult to control access to digital accounts and devices in the private context [35], not only based on a lack of technical knowhow of the users but also on very complex requirements regarding the granularity of access-control mechanisms [35]. However, exactly those finely adjustable access rights would be needed for the PHR-context, because the amount of information referring to the disease progression a patient or caregiver wants to get or wants to assign to others varies strongly from case to case [36]. This also poses a challenge for IT-developers, because they will need to focus on differently skilled software users and make patient portals convenient and attractive for all user groups. As the present results show, it is not self-evident for all potential users – especially in the caregiver’s role – that a patient might want to keep some PHR-content from family members. Therefore, it seems also necessary to inform all parties taking part in a PHR-scenario about rights and obligations – also in order to respect and protect their relationship of trust.

Generally, it is quite prevalent that discussions about the disease get avoided for example by couples affected by cancer [37]. Selman et al. (2015) showed that family dynamics might change in the context of fatal diagnoses such as cutaneous T-cell lymphoma, a rare and incurable form of cancer with a relatively long survival time [36]. These results are consistent with findings obtained in the presented study: on the one hand, the topic of how much information caregivers can cope with was brought up. On the other hand, it was made a subject of discussion that patients did not want to know a lot about their disease and treatment in order to reduce stress. As Pfister et al. (2017) showed, the idea of taking care of digital documents and information of an individual often causes stress in interview participants. In their study they dealt with Electronic Data Safes (EDS) serving as digital storing place for data as for example passwords and paperwork from insurances or banking as well as personal memorabilia (e.g. videos, photos) and examined who was involved in the management of such a digital legacy and what might be caused by this responsibility. They found that people mostly did not think about it a lot and, even if they considered it important, they tried to ignore and avoid this topic [38].

It should also be noted that the burden on cancer patients and thus the need for support from caregivers is particularly high. It would therefore be interesting to see how those affected by other diseases evaluate a proxy regulation.

### Limitations

Although patients from all over Germany and abroad are treated in the NCT, only people living in the region were selected for this study. Therefore, statements on transferability to a larger area should only be made with caution. While allowing for an in-depth qualitative study with rich and appropriate description of the phenomenon of interest, the small sample size implies that findings should be interpreted cautiously. It can be assumed that the innovative and technical character of this study attracted early adopters of ICT [39, 40], thus findings may not be generalizable to the regional colorectal cancer patient population. Although a convenience sampling strategy was applied, the study sample is heterogeneous regarding the educational level which might mitigate this limitation factor. Relatives could be present at the usability test and interview if requested by the participants. Thus, interviewees answers might have been influenced by the presence of their relatives. Caregivers were not included as study participants in the feasibility study, because the PEPA proxy account was not yet realized. Therefore, interviewed patients – like in the pre-implementation study – could merely hypothetically consider their caregivers’ role in a PEPA-usage scenario.

## Conclusions

In conclusion it can be said that there is a need to involve caregivers in PHR-usage in cancer care. A key finding is that the will to share data contained in a patient portal differs from case to case. Since the patient’s sovereignty over the data is a fact, there is a need for the patient to be able to easily sort which documents he/ she wants to share and which not. Therefore, a clear concept is needed on how a technical solution for representatives’ regulation can be implemented that is adaptable individually and easily to handle in everyday life.

Since the use of PHR in Germany still is not widespread, the opportunity occurs to rethink already established patient portal solutions elsewhere in order to better integrate caregivers. This might also help to foster user acceptance of PHR-patient portal solutions in general.

## Data Availability

The data supporting the findings of this study are not publicly available due to them containing information that could compromise research participant privacy.

## Abbreviations

EDS: Electronic Data Safe
GP: General Practitioner
ICD-10: International Statistical Classification of Diseases and Related Health Problems,10th Version
ICT: Information and communication technology
INFOPAT: Information Technology for patient-oriented health care in the Rhine-Neckar region
NCT: National Center for Tumor Diseases, Heidelberg, Germany
PEPA: Personal electronic health record developed within the INFOPAT project
PHR: Personal (electronic) health record
